# Bedtime regularity predicts positive affect among veterans with posttraumatic stress disorder: an ecological momentary assessment study

**DOI:** 10.1186/s12888-023-05373-9

**Published:** 2023-11-22

**Authors:** Jiyoung Song, Aaron J. Fisher, Steven H. Woodward

**Affiliations:** 1 Department of Psychology, University of California, 2121 Berkeley Way, Berkeley, CA 94720 USA; 2 Dissemination and Training Division, National Center for PTSD, 795 Willow Road, Menlo Park, CA 94025 USA

**Keywords:** Sleep, PTSD, Affect, Experience sampling, Actigraphy

## Abstract

**Background:**

Regularizing bedtime and out-of-bed times is a core component of behavioral treatments for sleep disturbances common among patients with posttraumatic stress disorder (PTSD). Although improvements in subjective sleep complaints often accompany improvements in PTSD symptoms, the underlying mechanism for this relationship remains unclear. Given that night-to-night sleep variability is a predictor of physical and mental well-being, the present study sought to evaluate the effects of bedtime and out-of-bed time variability on daytime affect and explore the optimal window lengths of over which variability is calculated.

**Methods:**

For about 30 days, male U.S. military veterans with PTSD (*N* = 64) in a residential treatment program provided ecological momentary assessment data on their affect and slept on beds equipped with mattress actigraphy. We computed bedtime and out-of-bed time variability indices with varying windows of days. We then constructed multilevel models to account for the nested structure of our data and evaluate the impact of bedtime and out-of-bed time variability on daytime affect.

**Results:**

More regular bedtime across 6–9 days was associated with greater subsequent positive affect. No similar effects were observed between out-of-bed time variability and affect.

**Conclusions:**

Multiple facets of sleep have been shown to differently predict daily affect, and bedtime regularity might represent one of such indices associated with positive, but not negative, affect. A better understanding of such differential effects of facets of sleep on affect will help further elucidate the complex and intertwined relationship between sleep and psychopathology.

**Trial registration:**

The trial retrospectively was registered on the Defense Technical Information Center website: Award # W81XWH-15–2-0005.

## Background

Sleep disturbances and consequent daytime impairment are common among individuals with posttraumatic stress disorder (PTSD) [1]. Up to 70% and 90% of patients with PTSD report experiencing nightmares and insomnia symptoms, respectively [2, 3], and the two sleep problems are reflected in the diagnostic criteria of PTSD in the *Diagnostic and Statistical Manual of Mental Disorders, 5th Edition* (*DSM-5*) [4]. Some studies have suggested that the severity of such sleep disturbances is associated with that of daytime dysfunction and that their presence in patients with PTSD can interfere with improvements in psychological symptoms in psychotherapy [5, 6]. Still, improvements in subjective sleep complaints have been shown to accompany improvements in PTSD symptoms [7], though it is important to note that sleep disturbances are among the most likely symptoms to remain at clinically significant levels after PTSD treatment [8].

Psychotherapeutic treatments have been shown to effectively treat trauma-related nightmares [9], reduce insomnia severity, and improve overall sleep quality among many patients with PTSD [10–12]. Among these treatments, cognitive behavioral therapy for insomnia (CBT-I) [13] is considered the first line treatment for insomnia and consists of cognitive restructuring, stimulus control, sleep restriction and compression, relaxation training, and psychoeducation in sleep hygiene [14]. Grounded on psychological and neurobehavioral findings demonstrating that irregular bedtime is associated with the development of insomnia [15, 16], CBT-I involves improving regularity in sleep–wake schedules, especially waketimes. Regular bedtimes can promote the synchrony between sleep drive and circadian rhythms to produce optimal sleep at night, whereas irregular bedtimes can lead to misalignment between said processes and poor sleep [17]. Qualitative research also supports this framework as individuals struggling with chronic insomnia cite variability in their night-to-night sleep as a source of frustration [18].

Regularizing sleep–wake schedules has been shown to optimize both subjective and objective sleep metrics. Irregular bedtimes have been shown to predict worse subjective sleep quality [19] as well as more severe daytime sleepiness [20]. In an experimental study, regularizing bedtimes resulted in lower sleep onset latency and increased sleep efficiency [21]. There are other physical health consequences of regularizing sleep behaviors [22]. Irregular sleep–wake schedules were found to predict worse self-reported health and higher rates of heart conditions and diabetes [23]. Mazzotti et al. [24] suggested that sleep patterns might even be linked to lipid metabolism and longevity in humans.

Bedtime regularity might be a sleep variable of particular interest for individuals with both mental health problems and sleep disturbances. Sleep–wake intraindividual variability has also been shown to predict greater depression severity among patients with bipolar disorder [25] as well as higher risk for bipolar disorder [26] and suicidality [27] among university students. In a study by Sánchez-Ortuño et al. [28], participants with a psychiatric diagnosis and comorbid insomnia exhibited greater sleep–wake variability than those with insomnia alone, suggesting the potential interaction between psychological symptoms and sleep disturbances. Similar observations were made for patients with PTSD and comorbid insomnia. In a study comparing patients with PTSD and sleep disturbances and those with insomnia alone, the two groups did not differ in their mean levels of total sleep time, sleep latency, and subjective insomnia ratings [29]. However, patients with both PTSD and sleep disturbances exhibited greater intraindividual variability than those with insomnia alone. Taken together, these findings reinforce the potential usefulness of evaluating night-to-night variability in sleep behaviors in patients with PTSD.

Longitudinal studies examining the impact of sleep among patients with PTSD in naturalistic settings have largely focused on the lag-one effects of sleep [30]. That is, mean levels of daytime affect and PTSD symptoms are compared to measures of previous night’s sleep, mostly self-reported duration and quality. For example, using ecological momentary assessment (EMA) surveys, DeViva et al. [31] found in a veteran sample that previous night’s shorter sleep duration predicted greater daytime PTSD symptom severity. Similarly, Short et al. [32] found that previous night’s poorer sleep quality and efficiency were associated with greater PTSD symptoms, less positive affect, and more negative affect the next day. The relationship between sleep quality and PTSD symptoms was found to be mediated by negative affect, indicating the importance of evaluating daytime affect as an outcome variable.

Calculating within-person variability for psychophysiological variables like sleep indices requires careful consideration of the window lengths over which variability is calculated [33]. For example, in a sample of older adults, a minimum of 7 days were found to be necessary to determine within-person variability of total sleep time, wake after sleep onset, sleep efficiency, and sleep fragmentation [34, 35]. Although within-person means over 3 days were comparable to those over 7 and 14 days, 3 days were not long enough to provide stable enough variability indices.

Although previous studies have demonstrated that bedtime regularity is associated with physical and mental health outcomes, no study to date, to our knowledge, has examined its impact on daytime affect in a sample of patients with PTSD. Given that regularizing bedtime is a core component of treating sleep disturbances that are common among patients with PTSD, it is important to evaluate the effects of bedtime regularity in addition to the well-studied effects of mean levels of sleep duration and quality. The present study using naturalistic longitudinal data had two primary aims. First, we sought to test whether bedtime and out-of-bed time regularity are significantly associated with daytime affect. We hypothesized that smaller bedtime and out-of-bed time variability in the past 7 days would be associated with greater daily positive affect and lower daily negative affect. We chose to examine the variability across 7 days based on Rowe et al.’s [34] finding that 7 days of measurement are sufficient to derive sleep variability indices. Second, we sought to explore the optimal number of days for the effects of bedtime and out-of-bed time regularity variability on daytime affect. That is, we were interested to find the number of days for which bedtimes and out-of-bed times needed to be regularized to have the biggest impact on daytime affect. The second aim was exploratory given the sparse literature related to the topic.

## Method

### Participants

The present study represents a secondary analysis of Woodward et al. [36]. The parent study compared affective experiences within subjects across weeks when participants were or were not accompanied by their service dog in a sample of veterans with PTSD in a residential treatment program. Results from the parent study indicated the association between the presence of a service dog and increased positive and reduced negative affect.

To be included in the parent study, participants (*N* = 64) needed to pass three levels of inclusion and exclusion criteria. First, they needed to obtain staff approval from a Veterans Affairs (VA) men’s residential trauma recovery program at the Menlo Park Division of the VA Palo Alto Health Care System. Staff members at the residential program assessed the participants for their engagement in residential PTSD treatment and fall risk. Psychotherapeutic components of the residential program included psychoeducation, treatment readiness, evidence-based treatments for PTSD, such as Cognitive Processing Therapy [37] and Prolonged Exposure [38]. Second, staff members at the service dog provider (Paws for Purple Hearts, Penngrove, CA) observed prospective participants over several 1-h sessions to ensure that they interact with the service dogs appropriately. Finally, the study team screened to exclude the participants for acute somatic disease, psychosis or mania, greater than mild traumatic brain injury, or medication with a β-adrenergic antagonist which could constrain heart rate, a variable of interest in the parent study. All participants provided written informed consent in accordance with the procedures of the Stanford/VA Palo Alto Health Care System Human Research Protection Program.

### Measures

#### Psychiatric assessment

Study team members used the Structured Clinical Interview for the *DSM-5* (SCID-5) [39], the Clinician Administered PTSD Scale for *DSM-5* (CAPS-5) [40] and the Brief Traumatic Brain Injury Screen (BTBIS) [41] to determine psychiatric diagnoses of the participants. The SCID-5 is considered the gold standard structured psychiatric interviewed designed to assess psychiatric diagnoses corresponding to diagnostic criteria outlined in *DSM-5* [4]. The CAPS-5 is a structured clinical interview that expands upon the *DSM-5* diagnostic criteria of PTSD with additional questions about the onset and duration of symptoms, distress, social and occupational functioning, recent improvement, and response validity. The BTBIS consists of three items screening for originating injury, acute alterations of consciousness, and persisting sequelae and has been judged “overly inclusive” in identifying traumatic brain injury [42]. We determined such bias to be appropriate for its use as an exclusionary screener in the parent study. In addition to the clinician-administered interviews and questionnaires, we employed the PTSD Checklist-5 (PCL-5) [43], the Combat Exposure Scale (CES) [44], and the Beck Depression Inventory II (BDI-II) [45]; as self-report psychometrics for purposes of sample comparison. Sound psychometric properties of the PCL-5 [46], the BDI-II [47], and the CES [48], including reliability, internal consistency, and convergent and divergent validity, have been established in the literature. Not all the measures collected in the parent study were examined in the present study.

#### Ecological momentary assessment

Participants received seven EMA survey notifications a day on their iPod Touch devices provided by the study team. At 7:00 am in the morning, they completed the postsleep report, and at 9:30 pm in the evening, they completed the presleep report. Between 7:00 am and 11:30 pm, they completed five surveys at quasirandom times within five 2.8-h windows. Within these windows, notification timing approximated a normal curve centered on the midpoint of the window such that participants rarely received two notifications in a short period. From the time participants received a survey notification, they had 1-h to complete the survey. This “interval-contingent” mode of EMA notifications was designed to capture the full signal of affect variation within the day, given the pragmatic consideration for participant burden of completing the surveys. Any missed surveys, including presleep and postsleep reports, were treated as missing data. The EMA surveys consisted of items based on the Positive and Negative Affect Schedule (PANAS) [49]. For example, the item assessing guilt read as follows: “To what extent have you felt guilty since your last report?” To reduce routinized responses, five positive affect and five negative affect items were randomly chosen from the parent set and presented in each EMA survey. Participants provided their responses on a 5-point Likert scale (1 = *not at all* to 5 = *extremely*) by moving a slider between the anchors. Each affect rating was time-stamped with 1-s accuracy. Participants who had a within-day survey completion rate of 80% or greater received five-dollar incentive payments.

#### Mattress actigraphy

The study team equipped participants’ beds in the residential treatment facility with mattress actigraphy. Mattress actigraphy is a nonintrusive, reliable, and valid way of capturing participants’ sleep behaviors via a mattress topper embedded with accelerometers that can be added to their regular mattresses [50]. Actigraphy data were obtained every night by a 1-inch cotton-covered latex foam mattress topper, which contained low-noise accelerometers (Silicon Designs, Issaquah, WA, Model 2210 2 g, bandwidth: 0–300 Hz) under the thorax. A bedside computer hosting a data acquisition circuit (Measurement Computing model 1408-FS, sampling rate, 600 Hz, amplitude resolution, 14-bits) recorded accelerometer signals, and custom software processed incoming accelerometer data into per 30-s time-series of heart rate, respiratory sinus arrhythmia, snoring, and body movement [50]. Bedtimes and out-of-bed times were determined using these accelerometer signals and recorded on the unit interval, where 0 indicated 12:00 am, and 1 11:59 pm. More detailed mattress actigraphy data acquisition processes are described in Woodward et al. [50].

### Procedure

Within the 2 weeks after admission, participants underwent psychiatric diagnostic assessments. They then waited about 6 weeks before they were matched with their service dog. The study team members oriented the participants to an iPod Touch (Apple, Inc., Cupertino, CA) and to the EMA app (esmi, Senti, Inc., San Francisco, CA). Once a week, participants participated in a laboratory session at the lab located near the residential program (cf. Woodward et al. [51]). During these weekly visits, study team members and participants reviewed together service dog contact, arousing events, and medication. Study staff also reminded participants to contact the study team between these laboratory sessions if they had any difficulties using their study device or app. More detailed study participants and procedure information can be found in Woodward et al. [36].

### Analytic plan

All analyses were conducted in R (Version 4.1.1) [52].

#### Daily affect

Within each EMA response, we averaged five positive and five negative affect items to estimate positive and negative affect for that observation, respectively. We then averaged all available positive and negative affect ratings in each day to compute daily positive and negative affect. Computing daily levels of positive and negative affect by averaging the respective affective items is a common practice in ecological studies examining the relationship between sleep and affect [53–55].

#### Bedtime and out-of-bed time variability

We used moving window approaches to compute a series of bedtime and out-of-bed time variability indices. For example, to calculate a bedtime regularity over 7 days, we calculated the standard deviation of bedtimes from the past seven days, which were expressed on the unit interval. Using this approach, we created bedtime and out-of-bed time variability indices based on previous 3–14 days from each participant’s longitudinal data.

#### Multilevel modeling

To account for the nested structure of our dataset, days within participants, we constructed and evaluated multilevel models using the *nlme* [56] and *lmerTest* [57] packages in R. Addressing both of our aims, we created a series of separate multilevel models where daily positive and negative affect were dependent variables, and a range of bedtime and out-of-bed time variability indices were the main predictors of interest. In every model, we covaried PTSD symptom severity at study start (CAPS-5 score), number of days since admission to the trauma residential program, and previous night’s actigraphic sleep efficiency (quiescent time divided by sleep period length). Covarying the number of days in the residential program was necessary to account for the effects of residential treatment and service dog on participants’ daily affective experiences. Because the participants were accompanied by their service dogs throughout the EMA portion of the study, the service dogs’ presence did not vary at the within-person level and thus was not included as a covariate in our models. From each model output, we calculated an effect size in Cohen’s *d* for the regression coefficient of bedtime or out-of-bed time variability index and compared them to determine the optimal number of days of which variability has the strongest association with daily affect. All our models included random intercepts and random slopes of time and variability indices. The *nlme* package defaults to maximum likelihood estimation to handle missingness in data.

## Results

### Sample characteristics and EMA engagement

Participants (*N* = 64) were all male (100%) due to the study design and majority White (56%). The group reported high PTSD symptom severity (*M* = 40.75, *SD* = 7.79). Participants usually went to bed at 10:50 pm (*SD* = 57.03 min) and got out of bed at 6:15 am (*SD* = 22.70 min). Average 7-day bedtime and out-of-bed time variabilities were 53.33 min (*SD* = 21.48 min) and 22.45 min (*SD* = 11.29 min), respectively. Participants’ out-of-bed times were likely constrained by residential treatment programming (i.e., breakfast). Average sleep efficiency was 0.85 (*SD* = 0.07). Participants’ average level of apnea–hypopnea index (AHI) fell in the mild range (*M* = 8.75, *SD* = 11.16). Participants responded to the EMA surveys for 30.19 days (*SD* = 13.79) and provided 210 observations (*SD* = 97.10). The mean response rate was 68% (*SD* = 20%). Following the recommendations by Jacobson [58], we did not set a strict compliance threshold and instead used all available observations in our models. Sample characteristics are presented in Table 1.

**Table 1:** Sample characteristics

Variable	*M* (*SD*) / *n* (%)
Age	37.31 (11.99)
Sex	
Female	0 (0)
Male	64 (100)
Ethnorace	
American Indian/Alaska Native	4 (6)
Asian	4 (6)
Black	4 (6)
Hawaiian/Pacific Islander	12 (19)
Non-White Hispanic or Latino	4 (6)
White	36 (56)
Sleep	
Bedtime	0.95 (0.04)
Out-of-bed time	1.25 (0.02)
7-day bedtime variability	0.04 (0.01)
7-day out-of-bed time variability	0.02 (0.01)
Sleep efficiency	0.85 (0.07)
Affect	
Positive	2.34 (0.59)
Negative	2.00 (0.64)
CAPS-5	40.75 (7.79)
AHI	8.75 (11.16)

The first four sleep variables are on the unit scale. For bedtime and out-of-bed time, 0 indicated 12:00 am, and 1 12:00 am the next day. For bedtime and out-of-bed time variability, 0 indicated 0 h, and 1 24 h

*CAPS-5* Clinician Administered PTSD Scale for *DSM-5*, *AHI* Apnea–hypopnea index

### Bedtime variability on affect

Lower bedtime variability in the past 7 days was significantly associated with greater daily positive affect (*B* = -3.55, *SE* = 1.32, *p* = 0.007, *d* = -0.20). Similarly, lower bedtime variabilities in the past 6 (*B* = -3.17, *SE* = 1.16, *p* = 0.007, *d* = -0.20), 8 (*B* = -3.46, *SE* = 1.50, *p* = 0.022, *d* = -0.17), and 9 (*B* = -3.53, *SE* = 1.73, *p* = 0.042, *d* = -0.15) days also significantly predicted greater daily positive affect. Bedtime variabilities over the other aggregate periods were not significantly associated with daily positive affect (all *p*s > 0.117).

**Fig. 1:**
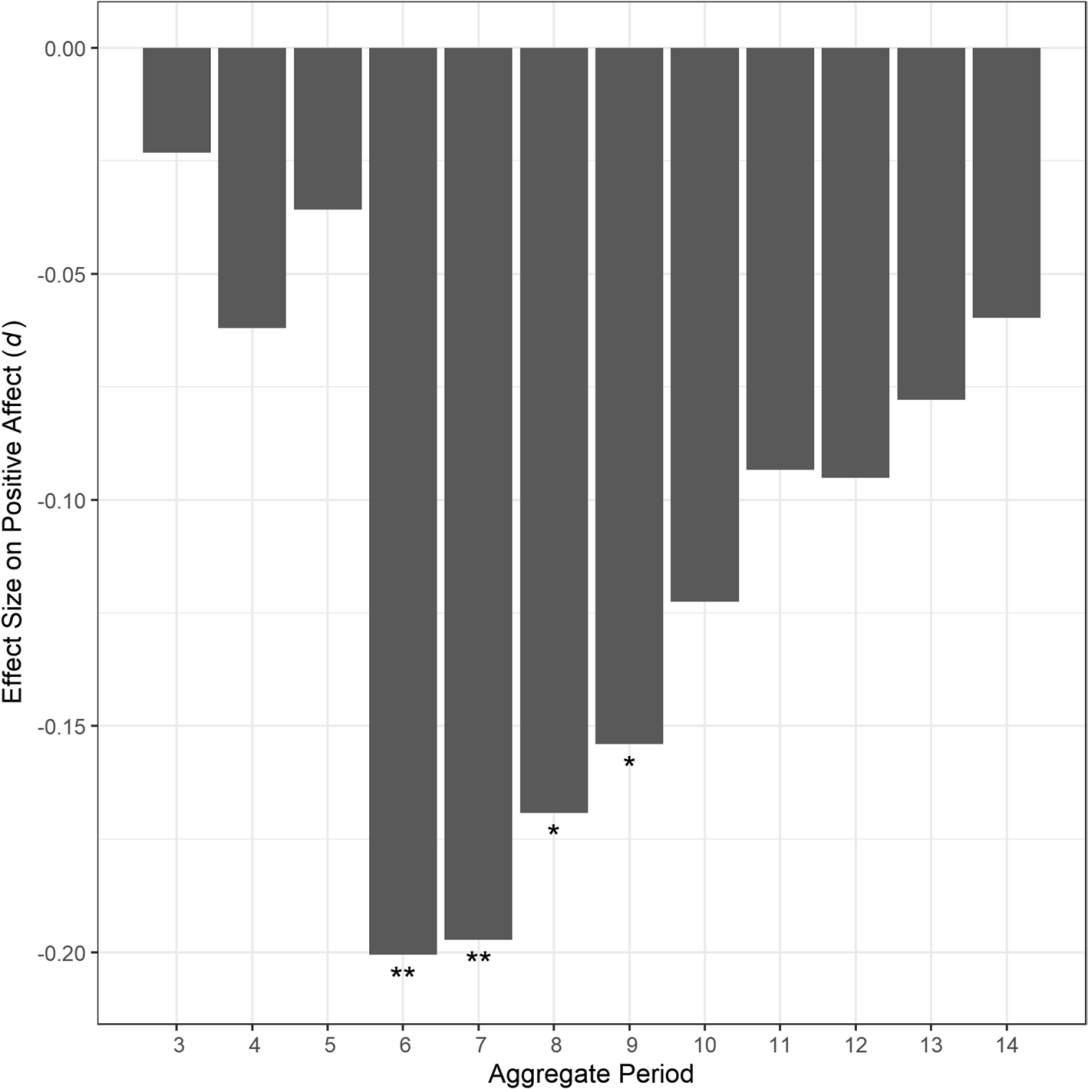
Effect Sizes of bedtime variability over 3–14 days on positive affect. Note. *d* = Cohen’s *d*. *p < .05. **p < .01

**Fig. 2:**
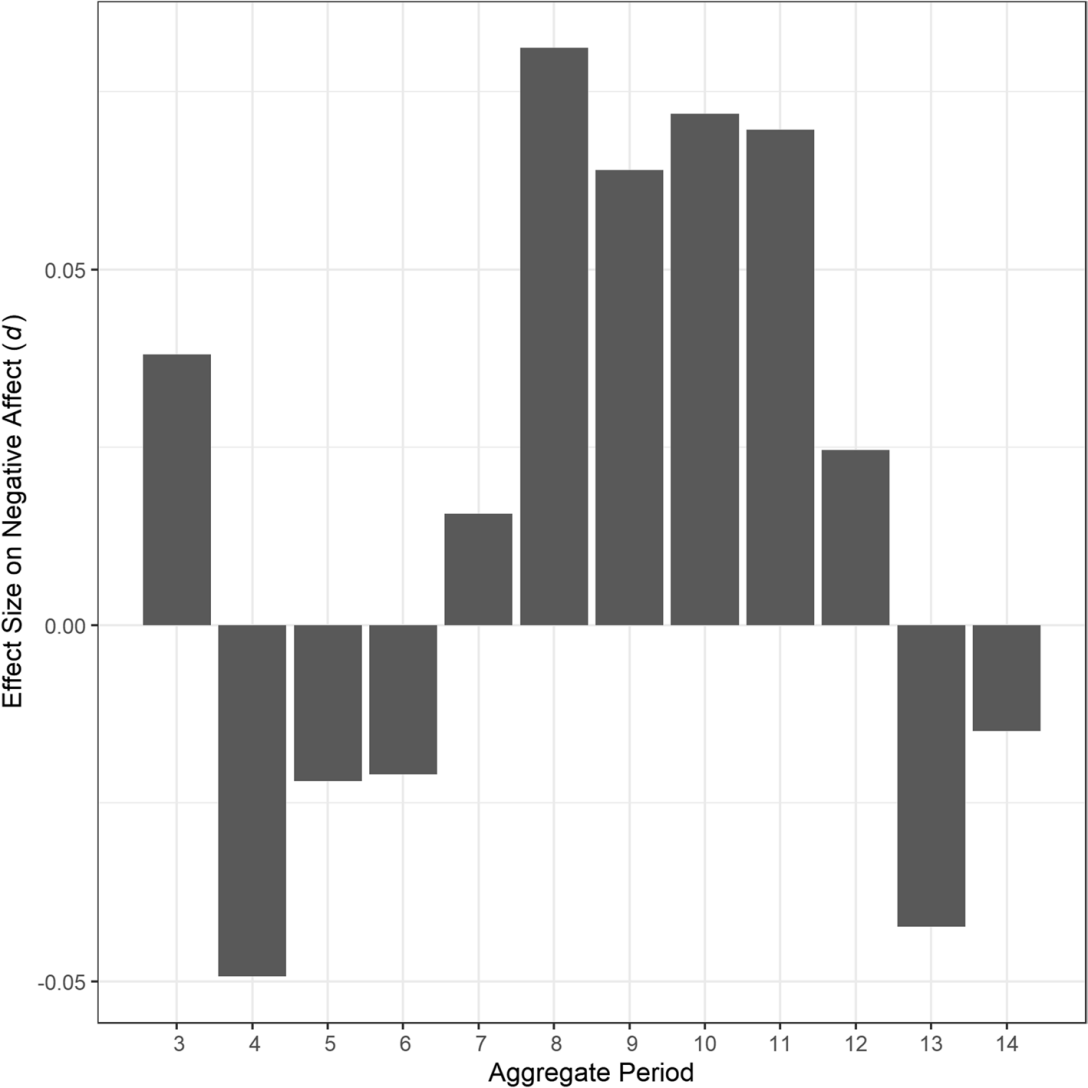
Effect sizes of bedtime variability over 3–14 days on negative affect. Note. *d* = Cohen’s *d*

Bedtime variability in the past 7 days was not significantly associated with daily negative affect (*B* = 0.28, *SE* = 1.32, *p* = 0.831, *d* = 0.02). There was also no significant association between bedtime variability and daily negative affect over the other aggregate periods (all *p*s > 0.270). Fixed effects of bedtime variability over 3–14 days on affect are presented in Table 2 and depicted in Figs. 1 and 2.

**Table 2:** Fixed effects of bedtime variability over 3–14 days on affect

Aggregate period	Positive affect					Negative affect				
	*B*	*SE*	*t*	*p*	*d*	*B*	*SE*	*t*	*p*	*d*
3	-0.32	1.04	-0.31	.757	-0.02	0.47	0.92	0.51	.612	0.04
4	-0.92	1.08	-0.85	.396	-0.06	-0.69	1.02	-0.67	.500	-0.05
5	-0.59	1.21	-0.49	.624	-0.04	-0.32	1.06	-0.30	.764	-0.02
6	-3.17	1.16	-2.73	.007	-0.20	-0.35	1.22	-0.29	.776	-0.02
7	-3.55	1.32	-2.69	.007	-0.20	0.28	1.32	0.21	.831	0.02
8	-3.46	1.50	-2.31	.022	-0.17	1.65	1.50	1.10	.270	0.08
9	-3.53	1.73	-2.04	.042	-0.15	1.44	1.71	0.85	.398	0.06
10	-2.96	1.89	-1.57	.117	-0.12	1.67	1.82	0.92	.359	0.07
11	-2.40	2.08	-1.15	.249	-0.09	1.66	1.93	0.86	.340	0.07
12	-2.53	2.22	-1.14	.255	-0.10	0.60	2.03	0.29	.768	0.02
13	-2.25	2.45	-0.92	.360	-0.08	-1.14	2.28	-0.50	.619	-0.04
14	-1.99	2.83	-0.70	.482	-0.06	-0.39	2.21	-0.18	.861	-0.01

*d* = Cohen’s *d*

### Out-of-bed time variability on affect

Out-of-bed time variability in the past 7 days was not significantly associated with daily positive affect (*B* = -0.66, *SE* = 2.46, *p* = 0.788, *d* = -0.02). There was also no significant association between out-of-bed time variability and daily negative affect over the other aggregate periods (all *p*s > 0.505).

**Fig. 3:**
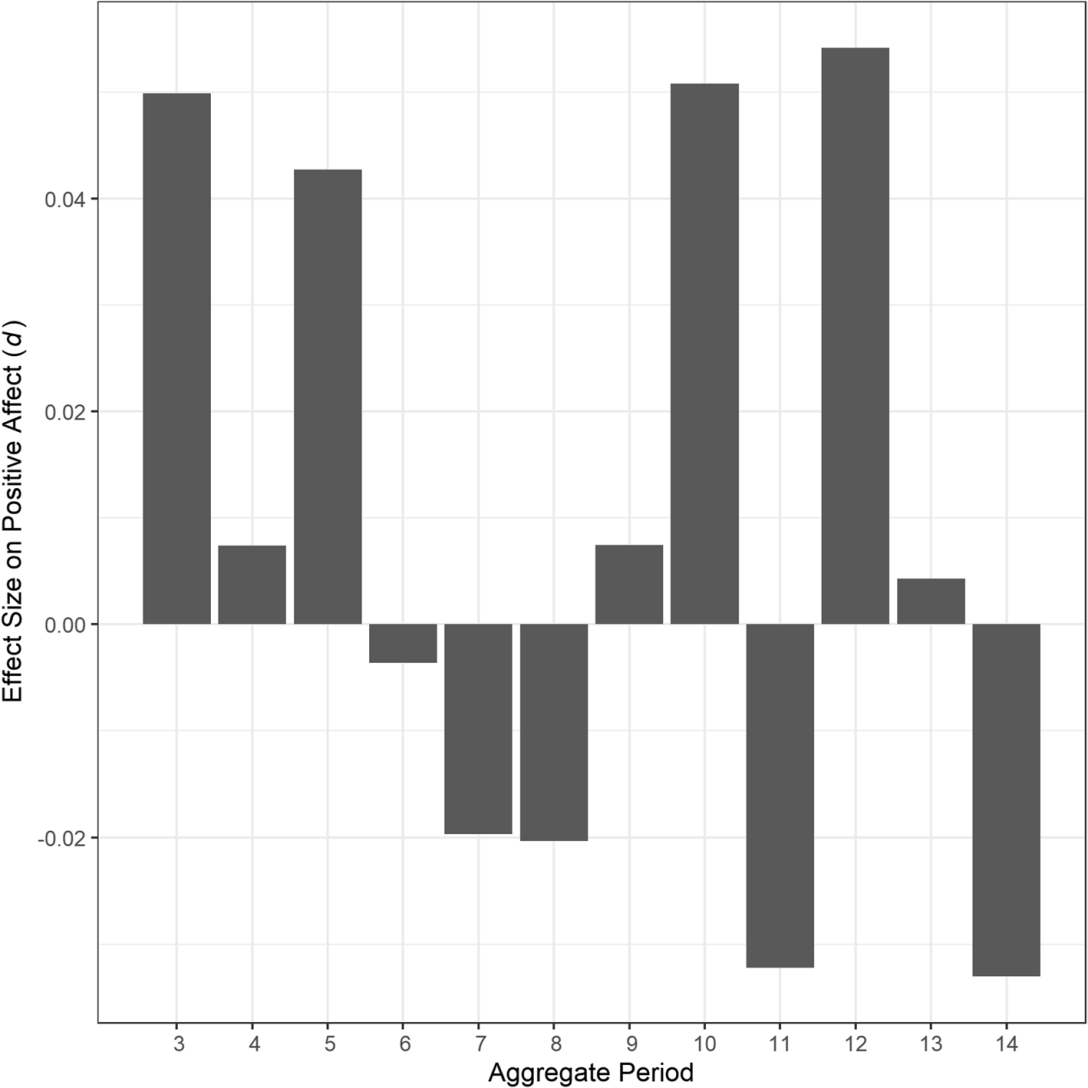
Effect sizes of out-of-bed time variability over 3–14 days on positive affect. *Note*. *d* = Cohen’s *d*

**Fig. 4:**
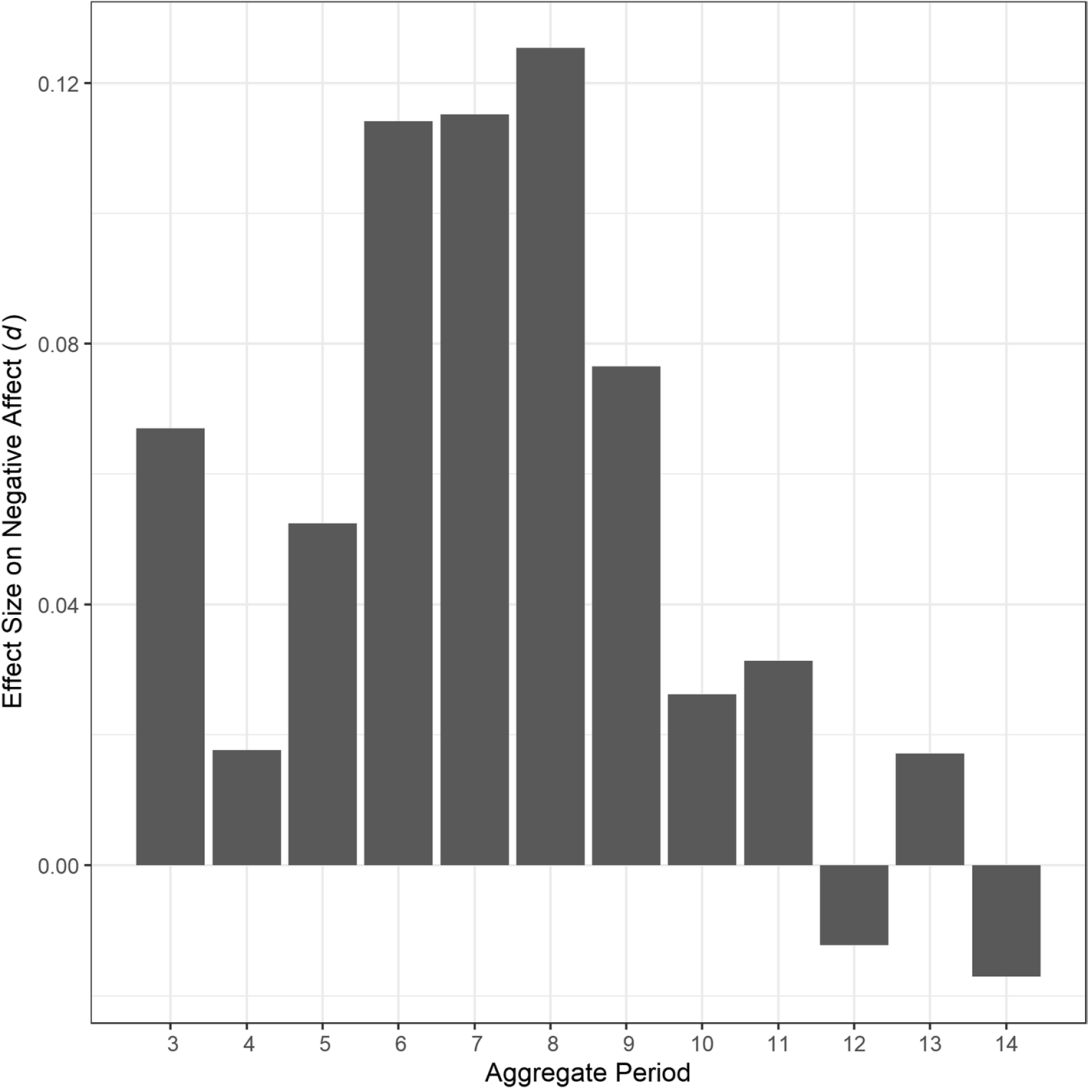
Effect sizes of out-of-bed time variability over 3–14 days on negative affect. *Note*. *d* = Cohen’s *d*

Out-of-bed time variability in the past 7 days was not significantly associated with daily negative affect (*B* = 4.75, *SE* = 2.79, *p* = 0.088, *d* = 0.13). There was also no significant association between bedtime variability and daily negative affect over the other aggregate periods (all *p*s > 0.117). Fixed effects of out-of-bed time variability over 3–14 days on affect are presented in Table 3 and depicted in Figs. 3 and 4.

**Table 3:** Fixed effects of out-of-bed time variability over 3–14 days on affect

Aggregate period	Positive affect					Negative affect				
	*B*	*SE*	*t*	*p*	*d*	*B*	*SE*	*t*	*p*	*d*
3	1.06	1.58	0.67	.505	0.05	1.45	1.62	0.89	.372	0.07
4	0.18	1.74	0.10	.919	0.01	0.44	1.83	0.24	.809	0.02
5	1.14	1.94	0.59	.558	0.04	1.53	2.14	0.72	.474	0.05
6	-0.11	2.27	-0.05	.961	0.00	3.66	2.36	1.55	.121	0.11
7	-0.66	2.46	-0.27	.788	-0.02	3.97	2.52	1.57	.117	0.12
8	-0.78	2.80	-0.28	.782	-0.02	4.75	2.79	1.71	.088	0.13
9	0.31	3.15	0.10	.922	0.01	3.21	3.17	1.01	.312	0.08
10	2.21	3.40	0.65	.515	0.05	1.09	3.25	0.33	.738	0.03
11	-1.40	3.53	-0.69	.691	-0.03	1.40	3.62	0.39	.699	0.03
12	2.54	3.91	0.65	.517	0.05	-0.52	3.57	-0.15	.884	-0.01
13	0.20	3.98	0.05	.960	0.00	0.92	4.58	0.20	.840	0.02
14	-1.66	4.25	-0.39	.697	-0.03	-1.01	5.05	-0.20	.841	-0.02

*d* = Cohen’s *d*

## Discussion

The present study evaluated the hypothesized effects of bedtime and out-of-bed time variability calculated over 7-night windows on daytime affect. Furthermore, we explored the optimal aggregate period for which bedtime and out-of-bed time variability has the largest effect on daytime affect. Male veterans diagnosed with PTSD undergoing a VA’s residential trauma recovery program completed PANAS-based EMA surveys seven times per day for about 30 days. Participants rated their positive and negative emotions in each EMA survey, and we averaged them at the day level to estimate daily positive and negative affect. Additionally, we extracted bedtime and out-of-bed time from mattress actigraphy embedded in participants’ beds. Using these longitudinal EMA and actigraphy data, we constructed multilevel models that accounted for the nested structure of our data (days within participants) to help answer our research questions.

Partially supporting our first hypothesis, when participants had a less variable bedtime in the past 7 days, they experienced greater positive affect on the subsequent day. Although bedtime variabilities over the 6, 8, and 9 days also had similar effects on positive affect, the aggregate periods of 6 and 7 days had the largest comparable effects. Interestingly, there was a jump in the effect of bedtime variability on positive affect between the aggregate periods of 5 and 6 days. One possible explanation is that irregular bedtimes in the past 5 days of fewer are not long enough to disrupt positive affect. Rather, the effect of regular bedtimes might need to build up for at least 6 days before enhancing positive affect, much like the harmful neuropsychological effect of sleep loss that builds over time [59–61]. The optimal aggregate periods of 6 and 7 days are also in line with previous findings that at least 6 days of actigraphy data are necessary for stable and meaningful sleep variability indices [34, 35]. On the other hand, the effect of regular bedtimes might diminish past the 9 days because the portion of the variance accounted by each night of sleep becomes too small. There likely exist individual differences in the optimal aggregate periods between 6 and 9 days, and the largest effect sizes of bedtime variability on positive affect over 6 and 7 days are reflective of the average fixed effect in our multilevel models.

In contrast to our hypothesis, participants’ variability in their bedtime over any aggregate periods did not significantly predict their subsequent daytime negative affect. This finding is especially interesting given the significant effects of bedtime variability on positive affect over the same aggregate periods. It is easy to assume positive and negative affect as simple mirror images of each other because they are reported to be highly negatively correlated in many self-report studies. However, the two can cooccur depending on situational stimuli [62] and are even said to be orthogonal [63, 64]. Indeed, the respective appetitive and defensive biobehavioral systems for positive and negative affect are not simple mirror images of each other in the brain [65, 66]. In the basolateral amygdala, for example, neurons have been shown to preferentially respond to appetitive and aversive stimuli [67]. There are also brain regions like the ventral hippocampus and nucleus accumbens exclusively dedicated for coding either appetitive and aversive behaviors that can influence positive and negative affective experiences [68]. Given that sleep indices have been shown to differently predict daily affect [69], bedtime regularity might represent one of such indices associated with positive, but not negative, affect.

Also, in contrast to our hypothesis, we did not observe any significant effects of out-of-bed time variability on affect. The differential association between sleep–wake schedule and affect is consistent with a previous finding that more regular bedtime, but not out-of-bed time, predicted lower daytime sleepiness [21], which can negatively influence mood at an extreme degree [70]. Similarly, lower bedtime variability, but not out-of-bed time variability, was associated with healthy lifestyle behaviors [71]. Another explanation for the absence of association is that night-to-night out-of-bed time variability was much smaller than bedtime variability. That is, participants stuck to their out-of-bed times about twice as regularly than their bedtimes, and such small variability in out-of-bed times was not enough to account for daily affect. Smaller out-of-bed time variability might also reflect more constraining factors for wake times, such as leaving for work in the general population [71] and participating in the residential treatment program in the study sample.

Although sleep treatments have been shown to improve not only sleep disturbances but also non-sleep PTSD symptoms [11], the exact underlying mechanisms remain unclear. Interestingly, our findings suggest that lowering night-to-night bedtime variability can promote greater daily positive affect. Positive affect might be of special interest in studying PTSD as it has been linked to the etiology of the disorder. Diminished positive affect in patients with PTSD might signal dysfunctional neural circuitry for reward processing activation, which in turn is thought to promote PTSD symptoms following a traumatic experience [72]. At the molecular genetic level, telomere shortening, which is present in several psychiatric disorders, has been shown to be associated with reduced positive, but not negative, affect [73]. Reduced positive affect was also found to be commonly present in individuals who had adverse childhood experiences, which are significant risk factors for PTSD [74]. Furthermore, positive affect constitutes a critical component in the resilience framework for conceptualizing and treating PTSD [75–77]. Taken together, these findings suggest that sleep treatments might target psychological symptoms in patients with PTSD by regularizing bedtimes and promoting positive affect.

### Limitations

Although the present study is the first to link bedtime regularity and daily affective experiences in a within-person design, some limitations need to be acknowledged. First, the study was conducted in a residential treatment program where participants might have experienced less pressure to fulfill work and family roles and thus less daily stress. Given that affect, stress, and sleep can influence one another [78], it is important to replicate our findings in a less controlled environment. Second, the routine schedule of the residential treatment program might have influenced the participants’ sleep–wake schedule. Though no strict constraints were imposed on the participants’ bedtimes and out-of-bedtimes, weak constraints could have arisen from bedtimes later than midnight attracting night staff’s clinical attention, roommates desiring earlier or later bedtimes, and breakfast being provided at the patient cafeteria. Still, the program did not schedule early morning or nighttime activities, and the sample exhibited considerable variability in their bedtime both at the between-person and within-person levels. Third, though the present study covaried for the number of days participants had been in treatment in all multilevel models, the session count was not included as a covariate. Given that the participants were receiving treatments for PTSD in a residential program, the number of sessions they had attended likely influenced their daily affective experiences. Similarly, the present study did not covary daily fluctuations in non-sleep and non-affect PTSD symptoms and their potential effects on sleep and affect. Future EMA studies should consider collecting PTSD severity for a better understanding of the daily dynamics between sleep behaviors, affective experiences, and PTSD symptoms, ideally in a sample where participants are not simultaneously receiving psychotherapy. Fourth, the sample exclusively consisted of male U.S. military veterans diagnosed with PTSD. It remains unclear whether the observed effects of bedtime variability on positive affect are generalizable to other populations. Since sleep disturbances are not exclusive to PTSD, it might be worth examining whether other populations will also experience added benefits of increased daily positive affect from regularizing their sleep–wake schedules.

## Conclusions

The present study provides novel evidence linking bedtime regularity to daily positive affect. It also offers a potential mechanism for mental health benefits of treatments for sleep disturbances in patients with PTSD. More precise understanding of the differential dynamics of multiple facets of sleep and affect will help further elucidate the intertwined nature of sleep and psychopathology beyond PTSD.

## Data Availability

The datasets used and/or analyzed during the current study are available from the corresponding author on reasonable request.

## Abbreviations

PTSD: Posttraumatic stress disorder
CBT-I: Cognitive Behavioral Therapy for Insomnia
EMA: Ecological momentary assessment
VA: Veterans Affairs
SCID-5: Structured Clinical Interview for the Diagnostic and Statistical Manual of Mental Disorders, 5th Edition
DSM-5: Diagnostic and Statistical Manual of Mental Disorders, 5th Edition
BTBIS: Brief Traumatic Brain Injury Screen
CAPS-5: Clinician Administered PTSD Scale for DSM-5
PCL-5: PTSD Checklist-5
CES: Combat Exposure Scale
BDI-II: Beck Depression Inventory II
PANAS: Positive and Negative Affect Schedule
